# High Fasting Blood Glucose Level With Unknown Prior History of Diabetes Is Associated With High Risk of Severe Adverse COVID-19 Outcome

**DOI:** 10.3389/fendo.2021.791476

**Published:** 2021-12-08

**Authors:** Wenjun Wang, Zhonglin Chai, Mark E. Cooper, Paul Z. Zimmet, Hua Guo, Junyu Ding, Feifei Yang, Xu Chen, Xixiang Lin, Kai Zhang, Qin Zhong, Zongren Li, Peifang Zhang, Zhenzhou Wu, Xizhou Guan, Lei Zhang, Kunlun He

**Affiliations:** ^1^ Key Laboratory of Ministry of Industry and Information Technology of Biomedical Engineering and Translational Medicine, Chinese People’s Liberation Army (PLA) General Hospital, Beijing, China; ^2^ Translational Medical Research Center, Chinese People’s Liberation Army (PLA) General Hospital, Beijing, China; ^3^ Medical Artificial Intelligence Research Center, Chinese People’s Liberation Army (PLA) General Hospital, Beijing, China; ^4^ Medical Big Data Center, Chinese People’s Liberation Army (PLA) General Hospital, Beijing, China; ^5^ Department of Diabetes, Central Clinical School, Faculty of Medicine, Nursing and Health Sciences, Monash University, Melbourne, VIC, Australia; ^6^ Department of Pulmonary and Critical Care Medicine, Chinese People’s Liberation Army (PLA) General Hospital, Beijing, China; ^7^ BioMind Technology, Zhongguancun Medical Engineering Center, Beijing, China; ^8^ China-Australia Joint Research Center for Infectious Diseases, School of Public Health, Xi’an Jiaotong University Health Science Center, Xi’an, China; ^9^ Artificial Intelligence and Modelling in Epidemiology Program, Melbourne Sexual Health Centre, Alfred Health, Melbourne, VIC, Australia; ^10^ Department of Epidemiology and Biostatistics, College of Public Health, Zhengzhou University, Zhengzhou, China

**Keywords:** COVID-19, FBG levels, diabetes, glycaemia control and treatment, complications

## Abstract

**Background:**

We aimed to understand how glycaemic levels among COVID-19 patients impact their disease progression and clinical complications.

**Methods:**

We enrolled 2,366 COVID-19 patients from Huoshenshan hospital in Wuhan. We stratified the COVID-19 patients into four subgroups by current fasting blood glucose (FBG) levels and their awareness of prior diabetic status, including patients with FBG<6.1mmol/L with no history of diabetes (group 1), patients with FBG<6.1mmol/L with a history of diabetes diagnosed (group 2), patients with FBG≥6.1mmol/L with no history of diabetes (group 3) and patients with FBG≥6.1mmol/L with a history of diabetes diagnosed (group 4). A multivariate cause-specific Cox proportional hazard model was used to assess the associations between FBG levels or prior diabetic status and clinical adversities in COVID-19 patients.

**Results:**

COVID-19 patients with higher FBG and unknown diabetes in the past (group 3) are more likely to progress to the severe or critical stage than patients in other groups (severe: 38.46% vs 23.46%-30.70%; critical 7.69% vs 0.61%-3.96%). These patients also have the highest abnormal level of inflammatory parameters, complications, and clinical adversities among all four groups (all p<0.05). On day 21 of hospitalisation, group 3 had a significantly higher risk of ICU admission [14.1% (9.6%-18.6%)] than group 4 [7.0% (3.7%-10.3%)], group 2 [4.0% (0.2%-7.8%)] and group 1 [2.1% (1.4%-2.8%)], (P<0.001). Compared with group 1 who had low FBG, group 3 demonstrated 5 times higher risk of ICU admission events during hospitalisation (HR=5.38, 3.46-8.35, P<0.001), while group 4, where the patients had high FBG and prior diabetes diagnosed, also showed a significantly higher risk (HR=1.99, 1.12-3.52, P=0.019), but to a much lesser extent than in group 3.

**Conclusion:**

Our study shows that COVID-19 patients with current high FBG levels but unaware of pre-existing diabetes, or possibly new onset diabetes as a result of COVID-19 infection, have a higher risk of more severe adverse outcomes than those aware of prior diagnosis of diabetes and those with low current FBG levels.

## Introduction

As of August 10, 2021, more than 204 million cases of coronavirus disease 2019 (COVID-19) have been reported worldwide, including more than 4.3 million COVID-19 related deaths (1). Patients with SARS-CoV-2 infection may develop severe complications, such as acute respiratory distress syndrome (ARDS) and multiple organ failures during hospitalisation, often resulting in deaths (2). Previous studies have established that COVID-19 patients with diabetes are more susceptible to inflammatory storms, leading to worse severe adverse clinical outcomes (2–4). These patients reportedly have a consistently higher risk of complications during hospitalisation, with more frequent mechanical ventilation, intensive care unit (ICU) admission, and deaths than otherwise (3–5). In particular, the odds of ICU admission are 36-fold higher when blood glucose levels increase from 5 to 10 mmol/L among COVID-19 patients (6, 17).

Numerous studies have reported that SARS-CoV-2 can infect pancreatic endocrine cells that express Angiotensin-converting enzyme 2 (ACE2), the well characterised receptor of this virus, thus directly affecting insulin production and glucose metabolism (7–11). SARS-CoV-2 infection is also known to stimulate the vast release of cytokines, thereby leading to hyperinflammatory syndrome and triggering an inflammatory cytokine storm (12). Furthermore, the cytokine storm is often associated with the dysregulation in glucose metabolism, manifested as diabetes mellitus or elevation in blood glucose level, leading to metabolic disorders (13). Indeed, common pro-inflammatory molecules, such as IL-6 and CRP, are involved in pathways leading to inflammatory cytokine storm as a result of SARS-COV-2 infection and in diabetes contributing to an aberrant glucose metabolism (14). Taken together, the interaction among overlapping pathways affected by COVID-19 and diabetes may synergise to trigger cytokine storms and adversely affect the prognosis of individuals with COVID-19 and diabetes.

Indeed, diabetes mellitus is a common comorbidity associated with deleterious clinical outcomes in COVID-19 patients (15). Some of these patients presented themselves with a newly diagnosed diabetes, showing an elevated FBG level without a prior history of diabetes. Firstly, these individuals might have had a pre-existing diabetes which was not previously diagnosed. Indeed, it was found in a survey in 2010 in China that ~70% of the Chinese participants living with diabetes had no diagnosis of diabetes, thus they did not know that they had diabetes (16, 17). Secondly, SARS-COV-2 infection is likely to cause new onset diabetes in those with no prior diabetes as well as increase the severity of pre-existing diabetes and pre-diabetes (9, 18–25), leading to elevation of blood glucose levels.

A number of recent studies have shown that COVID-19 patients with an elevated FBG level are at a higher risk of more severe COVID-19 than that seen in subjects with normal glucose levels (26–29). These studies have clearly shown that elevated glycemic levels and previous diagnosis of diabetes are associated with adverse and more severe outcome of COVID-19. More interestingly, new onset diabetes defined by an elevated glycemic level and a normal HbA1c level on admission has been shown to be associated with more severe outcome than those with pre-existing diabetes. However, it is unclear whether the COVID-19 patients who present with an elevated glycemic level on hospital admission, but were unaware of prior status of diabetes, have a higher risk of severe COVID-19 outcome than those who had a prior diagnosis of diabetes. In this study, we analysed the clinical outcomes of 2,366 COVID-19 patients in a major site of the initial COVID-19 epidemic, the Huoshenshan hospital, Wuhan, China, and specifically investigated whether the current FBG level and prior awareness of diabetic status can predict the risk of clinical complications, length of hospital stay, ICU admission, and death in COVID-19 patients.

## Methods

### Participants, Inclusion and Exclusion Criteria

We established a retrospective observational study cohort based on 3,059 COVID-19 patients admitted to the Huoshenshan hospital in Wuhan between February 04 and April 15, 2020. Subjects who had either a positive result of severe acute respiratory syndrome coronavirus two (SARS-Cov-2) detections in respiratory specimens by the reverse transcriptase-polymerase chain reaction assay or the presence of specific IgM and IgG antibodies to SARS-Cov-2 in serum were included in the study. We applied the following exclusion criteria on participants: 1. Subjects who had neither positive SARS-Cov-2 detection result nor anti-SARS-Cov-2 antibodies detected as specified above in the inclusion criteria. 2. Patients who were referred to other medical institutions during hospitalisation; 3. Patients who were admitted to the hospital multiple times; 4. Patients younger than 18 years old; 5. patients without historical records of diabetes diagnosis at admission and laboratory data of FBG within 72 hours after admission.

### Clinical and Outcome Indicators

Demographic, clinical, pre-existing comorbidities, laboratory test data, complications and outcome data were obtained from the hospital’s electronic clinical medical records. At admission, demographic, clinical, laboratory data and pre-existing comorbidities (coronary heart disease, cancer, chronic bronchitis, cerebrovascular disease, chronic kidney disease, chronic obstructive pulmonary disease, diabetes, hepatitis and hypertension) were collected within the first day after admission. Pre-existing comorbidities were defined according to the ICD10-CM code based on the patients’ clinical records and medical history (30).

We defined ICU admission as a key clinical outcome in this study. Clinical complications (acute respiratory distress syndrome, acute myocardial injury/heart failure, acute hepatitis/liver failure, respiratory failure, shock and acute kidney injury) and clinical outcomes (the length of stay, intensive care unit and death) were also collected during the hospital course from admission to the study endpoints. Detailed definitions for clinical symptoms and complications are provided in the supplemental materials section.

### Clinical Discharge Criteria

Patients had to meet all the following criteria before being discharged: (1) body temperature returned to normal (<37.5°C) for three consecutive days; (2) respiratory symptoms improved substantially; (3) pulmonary imaging showed an obvious resolution of inflammation; and (4) two consecutive laboratory tests showing negative detection of SARS-Cov-2 by RT-PCR, each at least 24 hours apart.

### Stratification of the Participants for Analysis

The subjects were stratified into 4 groups based on their FBG levels and awareness of prior status of diabetes. Group 1: patients with FBG<6.1mmol/L and unknown diabetes; Group 2: patients with FBG <6.1mmol/L and known diagnosis of prior diabetes; Group 3 patients with FBG≥6.1mmol/L and unknown diabetes; Group 4: patients with FBG≥6.1mmol/L and known diagnosis of prior diabetes. FBG 6.1 mmol/L was chosen as a cut point in stratification of subjects considering that FBG 6.1-6.9 mmol/L is defined to reflect impaired fasting glucose for Chinese population, from which the participants were recruited, according to the Guideline for the prevention and treatment of type 2 diabetes mellitus in China (2020 edition) (31), which is also consistent with the criteria of the World Health Organization.

### Statistical Analysis

We presented continuous variables as the median and interquartile range (IQR) and examined the differences among the groups using the Kruskal-Wallis one-way ANOVA. We presented categorical variables with the corresponding percentage and examined the differences using the χ2 or Fisher’s exact test. A multivariable cause-specific Cox proportional hazard model, adjusted for age, sex, chronic obstructive pulmonary disease, chronic kidney disease, cerebrovascular disease, coronary heart disease, cancer, chronic bronchitis, hepatitis and hypertension, was used to assess the associations between the FBG levels and prior diabetes status and ICU admission. Cumulative incidence curves were plotted to compare the incidence of ICU admission in 4 groups. A P-value of <0.05 was considered statistically significant. Statistical analyses were conducted using the R software (version 3.6.1).

## Results

### Demographic and Clinical Characteristics of Patients

**Figure 1:**
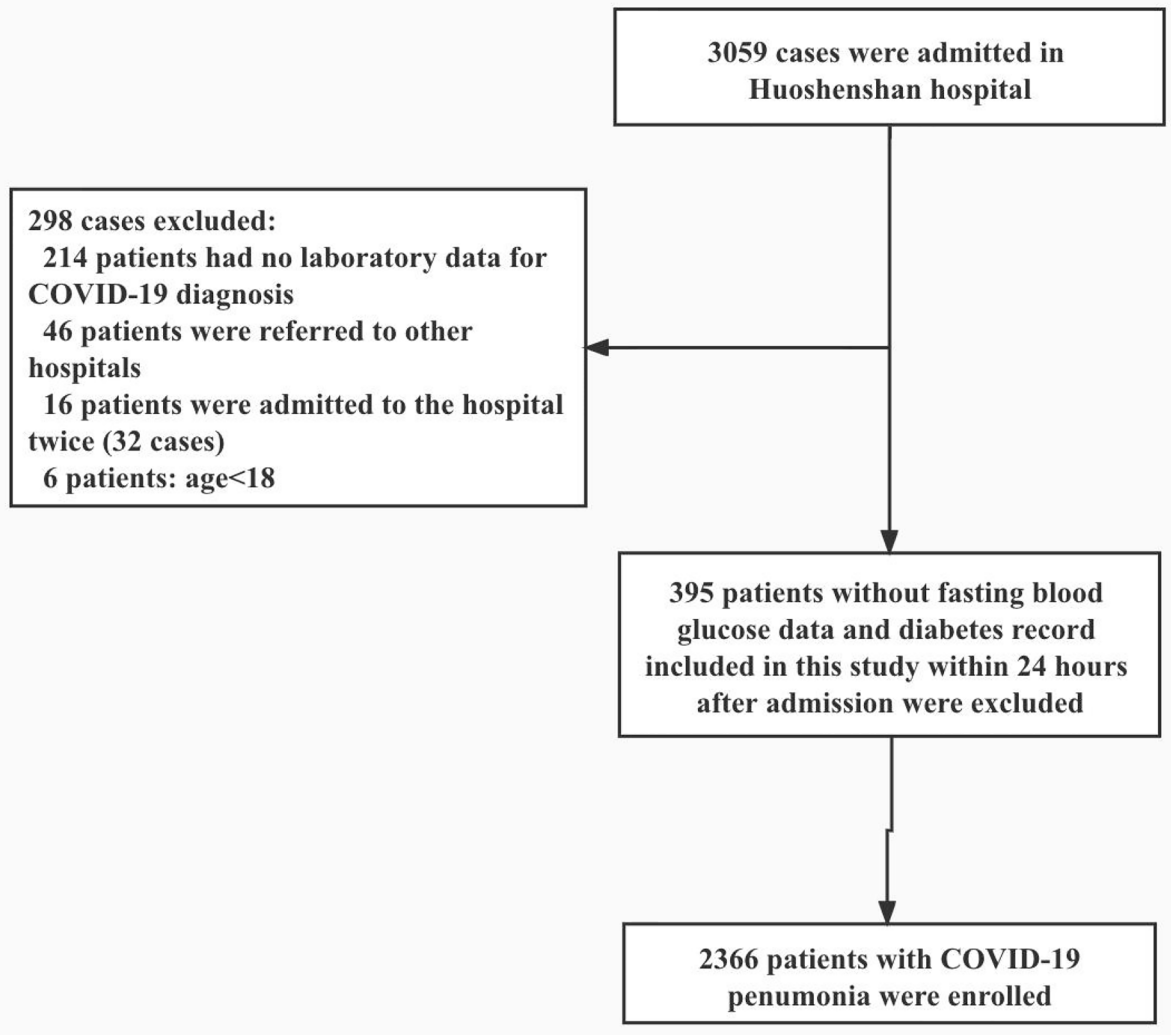
Flowcharts for the selection of study participants.

Of the 3,059 patients, we excluded 214 patients who were diagnosed as having COVID-19 based on clinical symptoms without laboratory tests, 46 patients who were transferred to other medical institutions, 16 patients who were admitted to the hospital multiple times, 6 patients who were <18 years of age. We further excluded 395 patients who had no record of FBG levels determined within 72 hours after admission or had no information to show whether they had a prior history of diabetes. A total of 2,366 COVID-19 patients were included for analysis (**Figure 1**).

The demographic characteristic, symptoms, pre-existing comorbidities, laboratory test results, complications and clinical outcomes are shown in **Table 1** for all the 4 stratified groups. The subjects who had <6.1 mmol/L FBG and had no record of prior diagnosis of diabetes at admission were stratified to Group 1. The subjects in this group had a significantly younger age than those in the other groups (median age: 58 years versus 63-65 years, Kruskal-Wallis one-way ANOVA, p<0.001) and had the lowest percentage of male participants (47.9% vs 53.4%-56.4%, p=0.051).

**Table 1:** Basic demographic characteristics, signs and symptoms, comorbidities, laboratory findings, treatment and clinical outcomes of patient groups in COVID-19. (Unit of FBG is mmol/L).

Variable	FBG<6.1 No prior diabetes Group 1	FBG<6.1 Diagnosed diabetes Group 2	FBG≥6.1 No prior diabetes Group 3	FBG≥6.1 Diagnosed diabetes Group 4	P-value
	(N = 1803)	(N = 101)	(N = 234)	(N = 228)	
**Demographic characteristics at admission**					
Age (yr)	58.00 (48.00,67.00)	63.00 (57.00,71.00)	64.00 (55.00,70.00)	65.00 (57.00,72.00)	<0.001**
					<0.001**
<45 —no. (%)	368 (20.41)	2 (1.98)	14 (5.98)	7 (3.07)	
45-59 —no. (%)	570 (31.61)	34 (33.66)	72 (30.77)	63 (27.63)	
60-74 —no. (%)	687 (38.10)	54 (53.47)	111 (47.44)	113 (49.56)	
>74 —no. (%)	178 (9.87)	11 (10.89)	37 (15.81)	45 (19.74)	
Male gender —no. (%)	864 (47.92)	57 (56.44)	125 (53.42)	125 (54.82)	0.051
Respiratory rate >20 min					
—no. (%)	490 (27.28)	25 (24.75)	91 (39.06)	86 (37.72)	<0.001**
Pulse rate >100 per min					
—no. (%)	276 (15.36)	17 (16.83)	44 (18.88)	44 (19.30)	0.282
Systolic blood pressure ≥140 mmHg					
—no. (%)	405 (24.49)	29 (31.52)	65 (29.41)	93 (45.15)	<0.001**
Diastolic blood pressure ≥90 mmHg					
—no. (%)	395 (23.90)	26 (28.26)	42 (19.00)	50 (24.27)	0.281
**Severity of COVID-19 patients —no. (%)**					<0.001**
Mild severity	24 (1.33)	0 (0.00)	0 (0.00)	0 (0.00)	
Moderate severity	1345 (74.60)	67 (66.34)	126 (53.85)	153 (67.11)	
Severe severity	423 (23.46)	30 (29.70)	90 (38.46)	70 (30.70)	
Critical severity	11 (0.61)	4 (3.96)	18 (7.69)	5 (2.19)	
**Signs and Symptoms —no. (%)**					
Fever	22 (1.23)	2 (1.98)	11 (4.72)	7 (3.08)	<0.0001**
Cough	1000 (55.5)	60 (59.4)	123 (52.6)	119 (52.2)	0.549
Fatigue	682 (37.83)	31 (30.69)	97 (41.45)	100 (43.86)	0.087
Diarrhea	50 (2.77)	1 (0.99)	10 (4.27)	3 (1.32)	0.198
Chest tightness	216 (11.98)	6 (5.94)	36 (15.38)	31 (13.60)	0.094
Shortness of breath	419 (23.24)	33 (32.67)	59 (25.21)	64 (28.07)	0.078
**Pre-existing comorbidities —no. (%)**					
Coronary heart disease	87 (4.83)	11 (10.89)	17 (7.26)	35 (15.35)	<0.0001**
Cancer	19 (1.05)	4 (3.96)	3 (1.28)	12 (5.26)	<0.0001**
Chronic bronchitis	42 (2.33)	2 (1.98)	6 (2.56)	1 (0.44)	0.246
Cerebrovascular disease	46 (2.55)	9 (8.91)	18 (7.69)	21 (9.21)	<0.0001**
Chronic kidney disease	33 (1.83)	6 (5.94)	5 (2.14)	11 (4.82)	0.004*
Chronic obstructive pulmonary disease	18 (1.00)	0 (0.00)	4 (1.71)	1 (0.44)	0.511
Hepatitis	24 (1.33)	1 (0.99)	3 (1.28)	3 (1.32)	0.993
Hypertension	435 (24.13)	57 (56.44)	86 (36.75)	131 (57.46)	<0.0001**
**Laboratory findings**				
Fasting blood glucose (mmol/L)	4.69 (4.38,5.09)	5.14 (4.78,5.68)	7.16 (6.57,8.44)	8.67 (7.19, 11.48)	<0.001**
C-reactive protein (mg/L)	1.73 (0.66,5.23)	2.19 (1.00,9.70)	8.52 (2.09,55.77)	3.67 (1.23,16.71)	<0.001**
D-dimer ( mg/L )	0.34 (0.18,0.66)	0.46 (0.24,0.74)	0.68 (0.39,1.64)	0.49 (0.28,1.03)	<0.001**
White blood cell count (10^9^/L)	5.60 (4.60,6.80)	5.50 (4.50,6.60)	6.30 (4.90,8.90)	6.30 (5.10,7.53)	<0.001**
Lymphocyte count (109/L)	1.53 (1.17,1.90)	1.53 (1.08,1.81)	1.15 (0.74,1.60)	1.44 (0.99,1.86)	<0.001**
Neutrophils count (10^9^/L)	3.33 (2.62,4.34)	3.31 (2.71,4.28)	4.36 (3.16,6.35)	3.73 (3.09,5.34)	<0.001**
Monocyte count (10^9^/L)	0.43 (0.34,0.54)	0.42 (0.35,0.53)	0.40 (0.29,0.56)	0.46 (0.37,0.57)	<0.001**
Lactate dehydrogenase (IU/L )	171.95 (148.80,205.10)	171.75 (150.97,203.03)	212.10 (175.30,296.40)	187.00 (159.72,243.15)	<0.001**
Thrombinogen time (s)	12.80 (12.23,13.46)	12.94 (12.40,13.65)	12.84 (12.23,13.87)	12.67 (12.06,13.52)	0.315
Total bilirubin (μmol/L)	9.50 (7.30,12.30)	9.80 (7.27,12.20)	9.80 (7.30,12.38)	9.40 (7.20,12.60)	0.967
Direct bilirubin (μmol/L)	3.30 (2.50,4.30)	3.45 (2.77,4.43)	3.60 (2.80,5.18)	3.50 (2.58,4.70)	0.001*
Albumin (g/L)	38.40 (35.60,40.70)	38.60 (36.15,40.90)	36.40 (32.73,39.00)	37.55 (34.55,40.40)	<0.001**
Alkaline phosphatase (IU/L)	68.30 (57.30,82.10)	70.50 (60.00,88.75)	78.40 (62.70,97.45)	75.10 (63.38,92.98)	<0.001**
Fibrinogen (g/L)	2.89 (2.57,3.28)	3.10 (2.88,3.46)	3.30 (2.80,3.82)	3.14 (2.80,3.50)	<0.001**
Creatinine (μmol/L)	64.10 (55.10,75.30)	64.60 (56.15,74.80)	65.00 (54.35,76.80)	62.90 (53.60,73.65)	0.433
Creatine kinase (U/L)	51.70 (37.50,72.45)	46.30 (34.25,75.30)	47.00 (33.70,75.50)	45.55 (33.62,67.92)	0.102
Creatine kinase-MB (IU/L)	8.40 (6.80,10.50)	9.00 (7.00,11.50)	9.50 (7.60,12.30)	9.50 (7.82,12.20)	<0.001**
Cystatin C (mg/L)	0.92 (0.82,1.05)	0.97 (0.87,1.08)	0.99 (0.84,1.15)	0.95 (0.84,1.10)	<0.001**
Platelets count (10^9^/L)	220.00 (182.00,268.25)	217.00 (184.00,255.50)	226.00 (178.00,290.00)	216.00 (177.00,260.25)	0.248
**Complications —no. (%)**					
Acute Respiratory distress syndrome	4 (0.22)	2 (1.98)	10 (4.27)	4 (1.75)	<0.001**
Acute myocardial injury/failure	21 (1.16)	3 (2.97)	9 (3.85)	4 (1.75)	0.012*
acute hepatitis/liver failure	25 (1.39)	2 (1.98)	9 (3.85)	6 (2.63)	0.034*
Respiratory failure	16 (0.89)	1 (0.99)	14 (5.98)	9 (3.95)	<0.001**
shock	6 (0.33)	1 (0.99)	5 (2.14)	2 (0.88)	0.009*
Acute kidney injury	3 (0.17)	1 (0.99)	2 (0.85)	1 (0.44)	0.045*
**Clinical outcomes**					
The length of stady (day)	12.00 (8.00,18.00)	13.00 (8.00,20.00)	17.00 (10.25,22.00)	14.50 (9.00,21.25)	<0.001**
ICU —no. (%)	39 (2.16)	4 (3.96)	33 (14.10)	16 (7.02)	<0.001**
Death —no. (%)	15 (0.83)	4 (3.96)	22 (9.40)	9 (3.95)	<0.001**

*P-value is between 0.05 and 0.001; **P-value < 0.001.

The subjects who had ≥6.1 mmol/L FBG and had no record of prior diagnosis of diabetes at admission were stratified to Group 3. This group had the highest proportion of patients with severe and critical clinical severity than that in other groups (38.5% vs 23.5-30.7% with severe clinical severity, and 7.7% vs 0.6-4.0% with critical clinical severity, p<0.001). Subjects in Group 3 had the highest levels of key inflammatory parameters among the four groups (p<0.001), such as C-reactive protein, D-dimer, neutrophils count, lactate dehydrogenase, direct bilirubin, fibrinogen, alkaline phosphatase, creatinine and cystatin C(**Table 1**). There were highest proportion of patients in Group 3 who had clinical complications, such as acute respiratory distress syndrome (4.3% vs 0.2-1.7%, p<0.001), acute myocardial injury/failure (3.8% vs 1.2-2.0%, p=0.012), acute hepatitis/liver failure (3.8% vs 1.4-2.6%, p=0.034), respiratory failure (6.0% vs 0.9-3.9%, p<0.001) and shock (2.1% vs 0.3-1.0%, p=0.009). People in Group 3 had the longest hospital stay (17 vs 12-14 days, p<0.001), the highest rate of ICU admission (14.1% vs 2.2-7.0%, p<0.001) and death (9.4% vs 0.8%, 4.0%, 4.0% in groups 1, 2 and 4, respectively; p<0.001).

### Risk of ICU Admission

Hazard ratio was analysed to show the relative risk of ICU admission, an indicator of critical severity of COVID-19 patients in various groups. People in Group 3 were found to have an >5 times higher risk of ICU admission during hospitalisation than group 1 (HR=5.38, 95% CI 3.46-8.35, P<0.001, **Table 2**). People in Group 4, like people in Group 3, had FBG≥6.1mmol/L on admission, but, unlike group 3, they knew that they had pre-existing diabetes. Interestingly, group 4, albeit showing higher odds than that seen in group 1, had lower odds than Group 3 (1.99, 1.12-3.52, P=0.019). Furthermore, the risk of ICU admission during hospitalisation was not significantly different (1.06, 0.38-2.98, P=0.915) between the 2 groups with <6.1mmol/L FPG, the Groups 1 and 2 regardless of their awareness of prior diagnosis of diabetes. After excluding the patients who required ICU admission within 24 hours after hospital admission, group 3 still demonstrated a significantly higher risk of ICU admission than group 1 (4.79, 1.98-11.59, P<0.001, **Table 2**). In contrast, group 4 did not show any statistically significant difference in risk of ICU admission, when compared to group 1 (1.46, 0.44-4.88, P=0.540).

**Table 2:** Adjust Hazard Ratio of FBG levels and diabetes to ICU admission in COVID-19 patients. (Unit of FBG is mmol/L).

Groups	HR^#^ (95%CI)	P-value	HR^##^ (95%CI)	P-value
FBG<6.1 & No prior diabetes (Group 1)	reference	reference	reference	reference
FBG<6.1 & Diagnosed diabetes (Group 2)	1.06 (0.38-2.98)	0.915	--	--
FBG≥6.1 & No prior diabetes (Group 3)	5.38 (3.46-8.35)	<0.001**	4.79 (1.98-11.59)	<0.001**
FBG≥6.1 & Diagnosed diabetes (Group 4)	1.99 (1.12-3.52)	0.019*	1.46 (0.44-4.88)	0.540

Adjust parameters: age, sex, pre-existing comorbidities (chronic obstructive pulmonary disease, chronic kidney disease, cerebrovascular disease, coronary heart disease, cancer, chronic bronchitis, hepatitis and hypertension); *Compared with the above reference the P-value is between 0.05 and 0.001; **Compared with the above reference, the P-value < 0.001; ^#^All COVID-19 patients; ^##^COVID-19 Patients were excluded by admitted to ICU at admission; Unit of FBG is mmol/L; ^--^HR is not analysed due to the number of this group patients is lower.

As a part of the sensitivity analysis, we additionally adjusted for demographic characteristics, symptoms, pre-exisiting comorbidities and laboratory indexes for the calculation of the Hazard Ratio. However, the findings of the adjusted model did not alter any of our conclusions (**Figures S1**, **S2** and **Table S1** in supplemental materials). Further detailed analyses and results were also provided in the supplemental materials.

### Cumulative Incidence Curve on the FBG Levels and Prior Diabetic Status to ICU Admission

**Figure 2:**
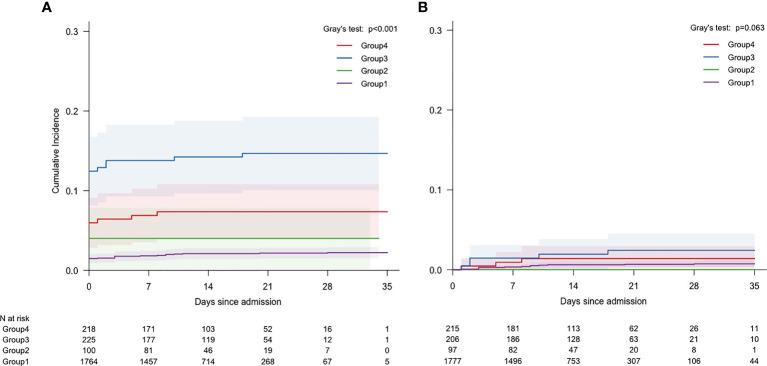
Cumulative incidence of ICU admission stratified by patients with FBG levels and diabetes: **(A)** All COVID-19 patients; **(B)** Remaining COVID-19 Patients after excluding those admitted to ICU on admission. (Unit of FBG is mmol/L).

**Figure 2** demonstrated the cumulative incidence of ICU admission events in all participants stratified by prior diabetic status and current FBG levels at hospital admission.

Group 3 had the highest risk of ICU admission at day 21 of hospitalisation. Its incidence was [14.1%, (9.6%-18.6%)], significantly higher than that of group 4 [7.0%, (3.7%-10.3%)], group 2 [4.0%, (0.2%-7.8%)] and group 1 [2.1%, (1.4%-2.8%)], (P<0.001, **Figure 2A**). After excluding the 71 patients who were admitted to ICU within 24 hours after hospital admission, group 3 still showed the highest risk of ICU admission at day 21 of hospitalisation. Its incidence was 2.4%, [0.1%-3.8%]). It is significantly higher than that seen in group 4 [1.4%, (0.0%-3.0%)], group 1 [0.7%, (0.3%-1.1%)] or group 2 [0.0%, (0.0%-0.0%)], (P=0.063) (**Figure 2B**).

## Discussion

Our study has evaluated whether FBG level in COVID-19 patients with or without awareness of the previous diagnosis of diabetes can predict an adverse outcome during hospitalisation. Our results demonstrate that patients who had a high FBG level (≥6.1mmol/L) at admission without a prior diagnosis of diabetes (group 3) have the highest risk of a severe or critical severity of COVID-19, such as a severe inflammatory response and multiple clinical complications. Patients in group 4 with pre-existing diabetes who presented with a high FBG level (≥6.1mmol/L) on admission have higher risk of higher severity of COVID-19 than those with a low FBG. In contrast, patients having a low FBG (<6.1 mmol/L) on admission had the lowest risk of clinical complications regardless of their awareness of the prior diagnosis of diabetes (groups 1 and 2). This finding of association between the elevated current FBG level and risk of adverse COVID-19 outcome is consistent with the previously reported findings (5, 6, 32, 33).

Interestingly, the patients in group 2, albeit having a prior diagnosis of diabetes, presented on admission with normalised FBG levels, indicating an adequate fasting glycaemic control. The group showed no significant differences in the proportion of patients developing clinically adverse outcomes compared to those from group 1, a group having no prior diagnosis of diabetes and presenting with a normal FBG level on admission. This result emphasizes a potentially important role of low current glycemic level, which could be a result of adequate glucose management in the past, in preventing a severe adverse outcome of COVID-19.

In contrast, the patients with uncontrolled FBG levels (≥6.1 mmol/L) and no prior diagnosis of diabetes (group 3) have the highest risk of an adverse prognosis, including a severe inflammatory response, having various clinical complications, long duration of hospitalisation, higher probablity of ICU admission and an increase in COVID-19 related death. This group of patients had a worse outcome than those in group 4 with pre-existing diabetes who had a similar high FBG on admission. This result is consistent with those from the previously reported studies where COVID-19 patients with a new onset diabetes, presented with hyperglycemia [(FBG >7mmol/L) and a normal HbA1c level (<5.6%)], are reported to have a worse COVID-19 outcome than those with pre-existing diabetes (27–29). Unlike this study, these previous studies did not examine the association between the unawareness of prior diabetes and the adverse outcome of COVID-19 in patients with high current FBG levels. In this study, the hyperglycaemic patients in group 3 had no awareness of their prior glycaemic status and there could be a significant proportion of patients in this group having pre-existing diabetes, considering a high prevalence and low diagnosis rate of diabetes as well as >50% adults having pre-diabetes in the general population in China (17). Inevitably, this group would also contain patients with new onset diabetes, as shown by another study on the same population in the same period of time where 21/82 (26%) COVID-19 patients had new onset diabetes with FBG ≥7.0 mmol/L and HbA1c <5.6% (28).

It has been observed that diabetes and/or current hyperglycaemia can be associated with deleterious outcome of COVID-19 (15, 33). The interaction between COVID-19 and diabetes is considered to be bi-directional (23). COVID-19 is also associated with increased severity of pre-existing diabetes leading to severe complications such as diabetic ketoacidosis (19–21, 34) as well as multi-organ injury (35, 36). Furthermore, a number of studies have shown that SARS-Cov-2 is able to infect multiple organs including those responsible for glucose metablolism, such as the pancreas leading to damage of pancreatic islets and impairment of β cell function (9, 25). Therefore, the elevation of glycemic level in the COVID-19 patients is not only possible to play a biological role leading to increased severity of Convid-19, it may also reflect the multi-organ injury as a result of SARS-Cov-2 infection, thus predicting a high risk of adverse outcome.

The specific identification of persons with diabetes at the time of COVID-19 diagnosis or those for the first time having an elevated FBG has not been part of the routine assessment of COVID-19 subjects. For example, the current Chinese guidelines for Diagnosis and Treatment Protocol for Novel Coronavirus Pneumonia (37) do not specifically address patients with diabetes or elevated FBG levels. The 2017 Chinese guidelines for the Prevention and Control of Type 2 Diabetes (38), which was published 2 years before the COVID-19 pandemic started, have not been amended to recommend specific treatment for COVID-19 patients. Bornstein et al. (22) have suggested that patients without diabetes, but have a higher risk for the metabolic disease should be monitored for new-onset diabetes triggered by COVID-19. Mary et al. (39) have developed a pragmatic approach for inpatient diabetes management and recommended insulin therapy for COVID-19 patients when their FBG level is above 10.00 mmol/L. This means, the patients without known diabetes but with FBG levels of ≥6.10 - <10.00 mmol/L, who are shown to be on risk (median 7.16 [IQR: 6.57, 8.44]) in this study, would not be specifically considered for their glycaemic management. Therefore, our finding provides evidence to suggest consideration of refining the clinical protocol to include this catogery of patients as on risk and eligible to receive glucose lowering management. Furthermore, the current World Health Organization guideline on drugs for COVID-19 strongly recommends the use of systemic corticosteroids in patients with severe and critical COVID-19 (40). Since previous studies demonstrate that corticosteroids can induce an elevation in blood glucose by promoting insulin resistance, further increasing the blood glucose level and the risk of type 2 diabetes (41, 42). Our findings suggest that these patients receiving corticosteroid treatment should be closedly monitored and carefully managed for their blood glucose in order to improve their prognosis. We consider the failure to provide timely glucose lowering management for patients with elevated FBG levels but not previously diagnosed diabetes as a missed opportunity to intervene to potentially improve the prognosis of these patients. This needs to be further confirmed in an intervention study.

Our findings further suggest that people with elevated FBG levels, particularly those without a previous diagnosis of diabetes, should be identified in the general population by FBG screening and considered as a prioritised group for COVID-19 vaccination in order to reduce the risk of COVID-19 infection, due to their higher risk of the severe adverse outcomes if being infected. However, it should be noted that this is solely based on the data from the hospitalized patients with overall severe disease. Whether this is applicable to the ambulatory patients in the general community should be further investigated. Nevertheless, at least 13 different COVID-19 vaccines have been registered by the WHO, and an estimated 11% of the world population has received at least one dose of the COVID-19 vaccine (43, 44). COVID-19 vaccines have been shown to be both effective and cost-effective in prevention new infections and severe COVID-19 diseases (45). The International Diabetes Federation strongly urges governments to prioritise vaccine access for people with diabetes (46). Based on our findings, we further propose to identify people with diabetes who have inadequately controlled FBG levels as well as to increase screening of the general population to identify those with elevated FBG levels, albeit they have not been previously diagnosed with diabetes. They should be prioritised as at risk subgroups for COVID-19 vaccination. The US CDC reported that among 86 million adults in the US who have prediabetes, 90% are not aware of their prediabetic status (47). In this population, 15-30% will progress to type 2 diabetes within five years (47). Even among adults with type 2 diabetes, only about a half have been diagnosed, according to the WHO (48) and the diabetes diagnosis rate can be much lower in some parts of the world, as shown by the Chinese survey several years ago (17). Hence, routine glucose screening at a population level to identify prediabetes or diabetes would be an important means to increase the awareness of individual glymaemic status, allowing them to receive adequate glucose lowering treatment to normalize their FBG level in order to reduce their risk of deleterious outcome if being infected by SARS-Cov-2. Equally important, these people can be identified for prioritising vaccination to be protected from infection, leading to a reduction of COVID-19-related mortality.

We appreciate there are several limitations to this study. Firstly, very few COVID-19 patients had HbA1c measured at Huoshenshan hospital; therefore, the average glucose control of the patients during the prior 3 months was unknown. The COVID-19 patients with uncontrolled FBG levels at admission cannot be diagnosed specifically as prediabetes, diabetes or COVID-19 associated new-onset diabetes. Secondly, our study only analyses FBG levels at three time points during the course of hospitalisation and may not fully reflect the variation of FBG levels. Thirdly, this cohort of COVID-19 patients was admitted to Huoshenshan hospital in the early phase of the pandemic, and the treatment options for COVID-19 and glucose management for elevated FBG may have been limited during the acute initial phase of the COVID-19 pandemic in Wuhan. Fourthly, this is a retrospective cohort study, which can only provide analytic results to show association among the current FBG levels, awareness of prior diabetes and outcome of COVID-19, and specifically designed intervention studies are required to establish a causal role for the current FGB level and/or awareness of individual’s prior glymcaemic status in an adverse outcome of COVID-19. Finally, we appreciate that obesity is recognized to be a potential risk factor for severe adverse outcomes in COVID-19. However, we don’t have obesity data for this cohort of patients at the early stages of the pandemic. We consider lack of obesity data for analysis to be a limitation in this study. Therefore, it would be worth considering additional cohort studies on patients with COVID-19 to specifically address the potential confounding issue in association with obesity.

## Conclusion

In conclusion, our study shows that COVID-19 patients with high FBG levels on admission with no awareness of their prior diagnosis of diabetes have the highest risk to develop severe inflammatory responses and complications of COVID-19 among patients with controlled FBG, including those with a known diagnosis of diabetes. Indeed, those with diagnosed diabetes but with FBG levels under control showed only a minimal risk of clinical adverse outcomes. COVID-19 patients presenting with an elevated FBG on admission with known diagnosis of prior diabetes would have a lower risk than those who were unaware of their prior glycaemic status. Thus, we recommended that there is a review of the current treatment guidelines for COVID-19 patients, emphasising glucose monitoring/awareness, optimising glycaemic management and prioritising COVID-19 vaccination for those who cannot obtain adequate glycemic control.

## Data Availability Statement

The raw data supporting the conclusions of this article will be made available by the authors, without undue reservation.

## Ethics Statement 

All methods of this study were carried out in accordance with relevant guidelines and regulations. This study was approved by Ethical Review of Scientific Research Projects of the Medical Ethics Committee of the Chinese PLA General Hospital, and obtained a waiver of one or more elements of informed consent form. The need for informed consent was waived by Medical Ethics Committee of the Chinese PLA General Hospital. The name of the ethics committee, The Medical Ethics Committee of the Chinese PLA General Hospital. The approval committee’s reference number, No. S2020-162-01.

## Author Contributions

Participants of the study should “contribute” as follows. WW, study design, data collection, data analysis, and writing. ZC, MC, PZZ, writing. HG, JD, FY, XL, XC, KZ, QZ, ZL, PFZ, ZW, data collection. XG, data collection and writing. LZ and KH, study design, data collection, data analysis, and writing. All authors contributed to the article and approved the submitted version.

## Funding

This work was supported by the Ministry of Industry and Information Technology of the People’s Republic China (2020-0103-3-1) and Key Technologies of Accurate Characterization and Comprehensive Evaluation of Military Body Fitness (19-163-12-ZD-037-003-02).

## Conflict of Interest

The authors declare that the research was conducted in the absence of any commercial or financial relationships that could be construed as a potential conflict of interest.

## Publisher’s Note

All claims expressed in this article are solely those of the authors and do not necessarily represent those of their affiliated organizations, or those of the publisher, the editors and the reviewers. Any product that may be evaluated in this article, or claim that may be made by its manufacturer, is not guaranteed or endorsed by the publisher.
