# Supplementary material for: High Fasting Blood Glucose Level With Unknown Prior History of Diabetes Is Associated With High Risk of Severe Adverse COVID-19 Outcome
Source: Front Endocrinol (Lausanne). 2021 Dec 8;12:791476. doi: 10.3389/fendo.2021.791476 (PMC8692378; doi:10.3389/fendo.2021.791476)
Supplement: Supplementary file 1 [file DataSheet_1.docx]

**Supplemental Appendix**

**Prioritising COVID-19 patients with elevated blood glucose levels for glycaemia control and treatment for complications**

Wenjun Wang^1-4^, Zhonglin Chai^8^, Mark E Cooper^8^, Paul Z Zimmet^8^, Hua Guo^5^, Junyu Ding^5^, Feifei Yang^1-4^, Xu Chen^1-4^, Xixiang Lin^1-4^, Kai Zhang^1^, Qin Zhong^1-4^, Zongren Li^1-4^, Peifang Zhang^10^, Zhenzhou Wu^10^, Xizhou Guan^5^, Lei Zhang^6-9^ **^#^**, Kunlun He^1-4^ **^#^**

# Authors contribute to supervision equally.

1. Key Laboratory of Ministry of Industry and Information Technology of Biomedical Engineering and Translational Medicine, Chinese PLA. General Hospital, Beijing, 100853, P.R.China.
2. Translational Medical Research Center, Chinese PLA. General Hospital, Beijing, 100853, P.R.China.
3. Medical Artificial Intelligence Research Center, Chinese PLA. General Hospital, Beijing, 100853, P.R.China.
4. Medical big Data Center, Chinese PLA. General Hospital, Beijing, 100853, P.R.China.
5. Department of Pulmonary and Critical Care Medicine, Chinese PLA General Hospital, Beijing, 100853, P.R.China.
6. China-Australia Joint Research Center for Infectious Diseases, School of Public Health, Xi'an Jiaotong University Health Science Center, Xi'an, Shaanxi, 710061, PR China.
7. Artificial Intelligence and Modelling in Epidemiology Program, Melbourne Sexual Health Centre, Alfred Health, Melbourne, Australia
8. Central Clinical School, Faculty of Medicine, Monash University, Melbourne, Australia
9. Department of Epidemiology and Biostatistics, College of Public Health, Zhengzhou University, Zhengzhou 450001, Henan, China.
10. BioMind Technology, Zhongguancun Medical Engineering Center, 10 Anxiang Road, 8th floor, Beijing, 100872, China

**Corresponding authors:**

**Kunlun He,** Key Laboratory of Ministry of Industry and Information Technology of Biomedical Engineering and Translational Medicine, Chinese PLA. General Hospital, Beijing, 100853, P.R.China.

[kunlunhe@301hospital.com.cn](mailto:kunlunhe@301hospital.com.cn)

**Lei Zhang,** China-Australia Joint Research Center for Infectious Diseases, School of Public Health, Xi'an Jiaotong University Health Science Center, Xi'an, Shaanxi, 710061, PR China. [Lei.zhang1@monash.edu](mailto:Lei.zhang1@monash.edu)

**一、Definition of clinical symptoms and complications**

1. Acute respiratory distress syndrome was defined as the acute onset of hypoxemia (PaO2/FiO2 ≤ 200) with bilateral infiltrates at x-rays and no evidence of left atrial hypertension [1].
2. Shock was defined as a clinical state of cellular and tissue gypoxia caused by reducing oxygen delivery and utilization or increasing oxygen consumption[2].
3. Acute heart failure was defined as a clinical syndrome characterized by typical symptoms (e.g. breathlessness, ankle swelling and fatigue) that may be accompanied by signs (e.g. elevated jugular venous pressure, pulmonary crackles and peripheral oedema) caused by a structural and/or functional cardiac abnormality [3].
4. Acute kidney injury was defined AKI was diagnosed by reference (exclusively) to SCr level, thus by an SCr increase ≥ 0.3 mg/dl (≥ 26.5 μmol/L) within 48 h, or an increase to ≥ 1.5-fold the baseline value, known or presumed to have developed within the prior 7 days [4].
5. Acute myocardial injury was defined as blood levels of cardiac biomarkers (hs-TNI) above the 99th-percentile upper reference limit, regardless of new abnormalities in electrocardiography and echocardiography[5].
6. Respiratory failure was defined as a failure to maintain adequate gas exchange and is characterized by abnormalities of arterial blood gas tensions [6].
7. Acute hepatitis was defined as a wide variety of clinical state characterized by elevated liver function indices caused by acute inflammation of hepatic parenchyma or injury to hepatocytes[7].
8. Acute liver failure was defined as severe acute liver injury with encephalopathy and impaired synthetic function (INR ≥ 1.5) in patients without pre-existing liver disease and with duration of < 26 weeks[8].
9. Cough, Fatigue, Diarrhea, Chest tightness and Shortness of breath symptoms of pneumonia were defined in accordance with the American Lung Association[9].
10. According to the Chinese guidelines for the prevention and control of type 2 diabetes (2017 edition)[10], we used the FBG level and diabetes as the key parameter to stratify the groups in our analysis. We categorized patients into 4 groups by their FBG level at admission, including patients with FBG<6.1mmol/L but yet diagnosed diabetes (group 1), patients with FBG<6.1mmol/L and diagnosed diabetes (group 2), patients with FBG≥6.1mmol/L but yet diagnosed diabetes (group 3) and patients with FBG≥6.1mmol/L and diagnosed diabetes (group 4).
11. The severity of COVID-19 was defined according to the Guidance 7^th^ edition. Patients were classified as ‘mild’ if there was no evidence of pneumonia on imaging nor any of the features for moderate or higher severity; as ‘moderate’ if they had evidence of pneumonia on imaging but no features of severe or higher severity; as ‘severe’ if they meet any of the following criteria: (1) respiratory distress (≥30 breaths/ min); (2) oxygen saturation ≤ 93% at rest on room air; (3) arterial partial pressure of oxygen (PaO2) or fraction of inspired oxygen (FiO2) ≦ 300mmHg (l mmHg=0.133kPa); and as ‘critical’ if they required mechanical ventilation, had a septic shock or required admission to ICU.

**二、Supplementary analysis**

**Methods**

**Statistical analysis**

We presented continuous variables as the median and interquartile range (IQR) and examined the differences among the groups using the Kruskal-Wallis one-way ANOVA. We presented categorical variables with the corresponding percentage and examined the differences using the χ2 or Fisher's exact test. Data imputation was performed if missing percentage < 30% using Multivariate Imputation by Chained Equations. Statistically significant variables were ranked and further selected using LASSO regression. The number of variables was defined as the number of variables when λ=λmin in LASSO. The set of variables were then included for the final multivariable cause-specific Cox proportional hazard model, which was used to assess the associations between the FBG levels and prior diabetes status and ICU admission. Cumulative incidence curves were plotted to compare the incidence of ICU admission in 4 groups. A P-value of <0.05 was considered statistically significant. Statistical analyses were conducted using the R software (version 3.6.1).

**Results**

**Risk of ICU admission**

Hazard ratio was analysed to show the relative risk of ICU admission, an indicator of critical severity of Covid-19 patients in various groups. People in Group 3 were found to have an ~3 times higher risk of ICU admission during hospitalisation than group 1 (HR=2.86, 95% CI 1.82-4.49, P<0.001, Figure S2A & Table S1). People in Group 4, like people in Group 3, had FBG≥6.1mmol/L on admission, but, unlike group 3, they knew that they had pre-existing diabetes. Interestingly, group 4, albeit showing higher odds than that seen in group 1, had lower odds than Group 3 (1.73, 1.14-2.64, P=0.011). Furthermore, the risk of ICU admission during hospitalisation was not significantly different (1.74, 0.71-4.62, P=0.25) between the 2 groups with <6.1mmol/L FPG, the Groups 1 and 2 regardless of their awareness of prior diagnosis of diabetes. After excluding the patients who required ICU admission within 24 hours after hospital admission, group 3 still demonstrated a significantly higher risk of ICU admission than group 1 (2.80, 1.12-7.03, P=0.028, Figure S2B & Table S1). In contrast, group 4 did not show any statistically significant difference in risk of ICU admission, when compared to group 1 (1.20, 0.35-4.18, P=0.77).

**Figure S1. The least absolute shrinkage and selection operator (LASSO) Cox proportional hazard model was used to validation selection of ICU admission. (A) LASSO coefficient profiles. (B) Tuning parameter (λ) selection in the LASSO model used 10, fold cross, validation via minimum criteria.**

**
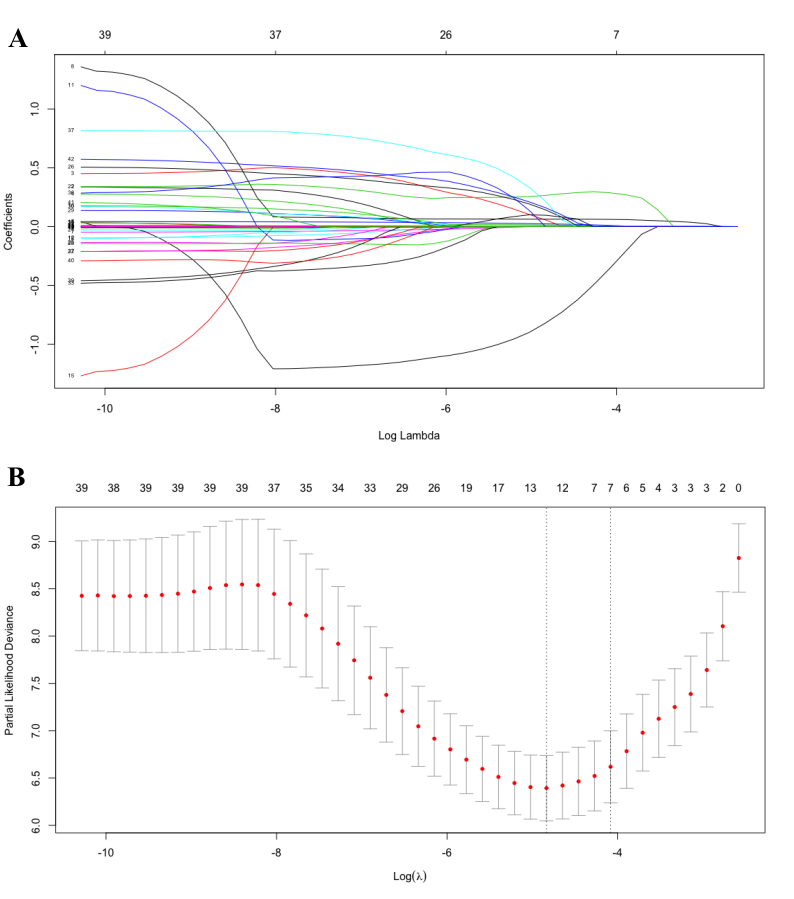
Figure S2 Multivariate cox proportional hazards regression for ICU admission in COVID-19 patients.**

(Figure 2 show multivariate cox regression analysis adjusted for selected variables, which were ranked and further selected using LASSO regression (Figure S1); *Compared with the above reference the P-value is between 0.05 and 0.001; **Compared with the above reference, the P-value < 0.001; A: All COVID-19 patients; B: COVID-19 Patients were excluded by admitted to ICU at admission; --HR is not analysed due to the number of this group patients is lower.)

**
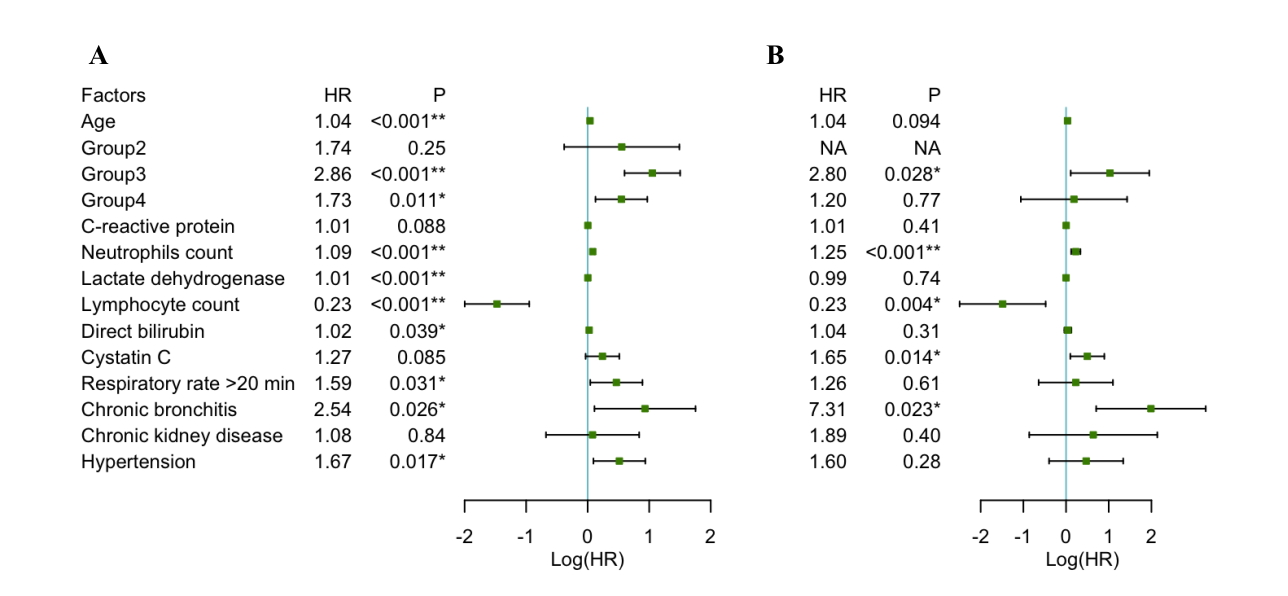
Table S1 Adjust Hazard Ratio of FBG levels and diabetes to ICU admission in COVID-19 patients. (Unit of FBG is mmol/L)**

| Groups | HR^#^ (95%CI) | P-value | HR^##^ (95%CI) | P-value |
| --- | --- | --- | --- | --- |
| FBG<6.1 & No prior diabetes (Group 1) | reference | reference | reference | reference |
| FBG<6.1 & Diagnosed diabetes (Group 2) | 1.74(0.71-4.62) | 0.25 | -- | -- |
| FBG≥6.1 & No prior diabetes (Group 3) | 2.86(1.82-4.49) | <0.001** | 2.80(1.12-7.03) | 0.028* |
| FBG≥6.1 & Diagnosed diabetes (Group 4) | 1.73(1.14-2.64) | 0.011* | 1.20(0.35-4.18) | 0.77 |

Table 2 the HR adjusted for selected variables, which were ranked and further selected using LASSO regression (Figure S1 in supplemental materials); *Compared with the above reference the P-value is between 0.05 and 0.001; **Compared with the above reference, the P-value < 0.001; #: All COVID-19 patients; ##: COVID-19 Patients were excluded by admitted to ICU at admission; Unit of FBG is mmol/L; ^--^HR is not analysed due to the number of this group patients is lower.

**Reference**

1. WHO. Clinical management of severe acute respiratory infection when Novel coronavirus (nCoV) infection is suspected: interim guidance. . Jan 11, 2020.

2. Gaieski DF, Mikkelsen M. Definition, classification, etiology, and pathophysiology of shock in adults. UpToDate, Waltham, MA Accesed 2016; 8: 17.

3. Ponikowski P VA, Anker SD, et al. . 2016 ESC Guidelines for the diagnosis and treatment of acute and chronic heart failure: The Task Force for the diagnosis and treatment of acute and chronic heart failure of the European Society of Cardiology (ESC) Developed with the special contribution of the Heart Failure Association (HFA) of the ESC. . Eur Heart J 2016;37:2129-2200.

4. KDIGO clinical practice guideline for acute kidney injury. . March, 2012.

5. Gao C WY, Gu X. . Association between cardiac injury and mortality in hospitalized patients infected with avian influenza A (H7N9) virus. . Crit Care Med 2020

6. Lotano R. Nonpulmonary Causes of Respiratory Failure. Critical Care Medicine (Third Edition) 2008.

7. Schaefer TJ JS. Acute Hepatitis. Treasure Island (FL): StatPearls Publishing, Updated 2020 Oct 27.

8. Lukashyk SP, Karpov IA. ACUTE LIVER FAILURE IN ADULTS: ETIOLOGY, CLINICAL MANIFESTATIONS, METHODS OF CORRECTION. The Russian Archives of Internal Medicine 2017; 7(3): 171-80.

9. Prevention CfDCa. Symptoms of COVID-19. Available at: <https://www.cdc.gov/coronavirus/2019-ncov/symptoms-testing/symptoms.html>. Accessed April 14.

10. Society CD. Guidelines for the prevention and control of type 2 diabetes in China (2017 Edition). April 2018.
